# Arthroscopic ankle fusion only has a limited advantage over the open operation if osseous operation type is the same: a retrospective comparative study

**DOI:** 10.1186/s13018-020-01599-5

**Published:** 2020-02-26

**Authors:** Chenggong Wang, Can Xu, Mingqing Li, Hui Li, Long Wang, Da Zhong, Hua Liu

**Affiliations:** 1 Department of Foot and Ankle Surgery, Xiangya Hospital Central South University, No.87 Xiangya Road, Changsha, 410008 Hunan China; 2 Department of Orthopedics, Xiangya Hospital Central South University, No.87 Xiangya Road, Changsha, 410008 Hunan China

**Keywords:** Arthroscopic ankle fusion, Open ankle fusion, Selection bias, Follow-up, Questionnaire survey

## Abstract

**Background:**

A great deal of research suggests that arthroscopic ankle fusion (AAF) has advantages over open ankle fusion (OAF), but these outcomes would be imprecise because of a selection bias. The purpose of this study is to verify which is better for ankle fusion, AAF or OAF. We regrouped the OAF group into two subgroups according to whether the osseous operation type is the same as AAF group. The goal is to minimize the impact of disease severity, thereby reducing selection bias to some extent.

**Methods:**

We retrospectively analyzed the data of ankle fusion in our hospital between July 2015 and October 2018. Forty-three patients were enrolled and divided into AAF group (*n* = 17) and OAF group (*n* = 26). In order to eliminate selection bias, we divided OAF group into complex osseous operation subgroup (COO subgroup) (*n* = 15) and simple osseous operation subgroup (SOO subgroup) (*n* = 11). The osseous operation type of SOO subgroup is the same as AAF group. Then, we compared the differences between these groups. All patients were followed up at least 1 year after operation. We analyzed data, including etiology composition, surgical time, intra-op blood loss, reduction of albumin, total hospital stays, union time, fusion situation, complications, radiological examination, functional score, and questionnaire survey. Then we performed statistical analyses.

**Results:**

We found that the etiological components of AAF group and OAF group were different; the etiological components of AAF group and SOO subgroup were similar. We found that AAF group has advantages over OAF group and COO subgroup in general. However, except in terms of surgical trauma, hospital stays, and short-term complications occurred, the AAF group has not obvious advantages over SOO subgroup, including intra-op blood loss, fusion condition, postoperative function score, and postoperative patient satisfaction; and AAF group need more surgical time than the SOO subgroup.

**Conclusions:**

The arthroscopic ankle fusion can bring a good curative effect; however, if the osseous operation type is the same, the arthroscopic ankle fusion only has a limited advantage over the traditional open operation in perioperative soft tissue protection and enhanced recovery after surgery.

## Background

Ankle fusion is a reliable and effective option in the treatment of end-stage ankle arthritis and pain [1]. While ankle fusion carries a high rate of union, the optimal surgical method continues to be debated with more than 40 techniques described in the literature [2–6]. Although most open operation methods have achieved a good curative effect, they have however been associated with many complications [7, 8]. These complications of open ankle fusion resulted in the development of less invasive techniques such as arthroscopic ankle fusion [9]. A great deal of research suggests that arthroscopic ankle fusion has advantages over open operation such as faster time to union, lower morbidity, lower blood loss, faster rehabilitation, and shorter hospital stay [10–14]. Many scholars believe that the reason of these advantages by arthroscopic ankle fusion probably because periosteal stripping is not necessary, and the local circulation remains intact, creating a more favourable environment for fusion to occur [15].

However, most of these studies were case series report, only a few of them were comparative studies; in addition, these comparative studies usually ignored the influence of two aspects on the results. First, there are a number of etiologies for the patients need ankle fusion, and the disease conditions are difference for the patients. The different etiologies and disease conditions will affect the choice of operation method. In fact, some patients need complex osseous operation with poor curative effect could only choose open surgery and the comparison results are also likely to favor arthroscopic ankle fusion method; such as distal fibula was removed, need more than 8 cm^3^ impacted bone graft, need structural bone graft, even tibia-talus-calcaneus fusion. As a matter of fact, such selection bias [16] can hardly be avoided in the study of the surgical effect of ankle fusion. Second, ankle fusion is not a perfect surgical method in a sense, because AOFAS scores [17] of almost all ankle fusion patients cannot exceed 85 [18]. Therefore, the judgment of the efficacy of ankle fusion should be combined with subjective evaluation, which was also lacking in relevant studies.

So we conducted a retrospective comparative study to determine whether arthroscopic ankle fusion (AAF) or open ankle fusion (OAF) was more effective. On the one hand, we regrouped the OAF group into complex osseous operation subgroup (COO subgroup) and simple osseous operation subgroup (SOO subgroup) based on osseous operation type; the SOO subgroup condition was similar to AAF group, which excluded the cases whose osseous operation types were complex or excessive, and could not be available in AAF cases; the goal is to minimize the selection bias to some extent. On the other hand, we innovatively used questionnaire during follow-up, which referred to Musculoskeletal Outcomes Data Evaluation and Management Scale (MODEMS) questionnaire [19]. To the best of our knowledge, this is the first report to use MODEMS questionnaire in study of ankle fusion. It is worth noting that some different results seemed to be found unlike most studies that have been done.

## Method

### Study design

This study has been approved by the Ethics Committee of our hospital. We retrospectively analyzed a series of data of patients with ankle fusion of our department between July 2015 and October 2018. Data were obtained from the registration system of foot and ankle surgery department, the medical record information system, and the follow-up system of patients’ service center of our hospital. All patients included in this retrospective study were all agreed to participate in the study and have been signed written consent. All cases were operated by professor Liu, who has a wealth of experience of open ankle fusion and arthroscopic ankle fusion; in addition, he is the only expert surgeon in our hospital and has the largest surgical amount of arthroscopic ankle fusion in Hunan province in China. The inclusion criteria were as follows: (1) primary ankle fusion surgery; (2) unilateral ankle fusion; (3) Takakura staging was worse than IIIA [20] or AOFAS score was less than 50; and (4) voluntary provision of medical records to this study. The exclusion criteria were as follows: (1) refused to participate in this study; (2) ankle fusion by using external fixator; (3) the homolateral lower limb includes other operations, such as joint arthroplasty, internal fixation, tibia-talus-calcaneus fusion ,or other joint fusion; (4) physical activity disorders caused by other disease, such as stroke; and (5) mental illness.

**Fig. 1:**
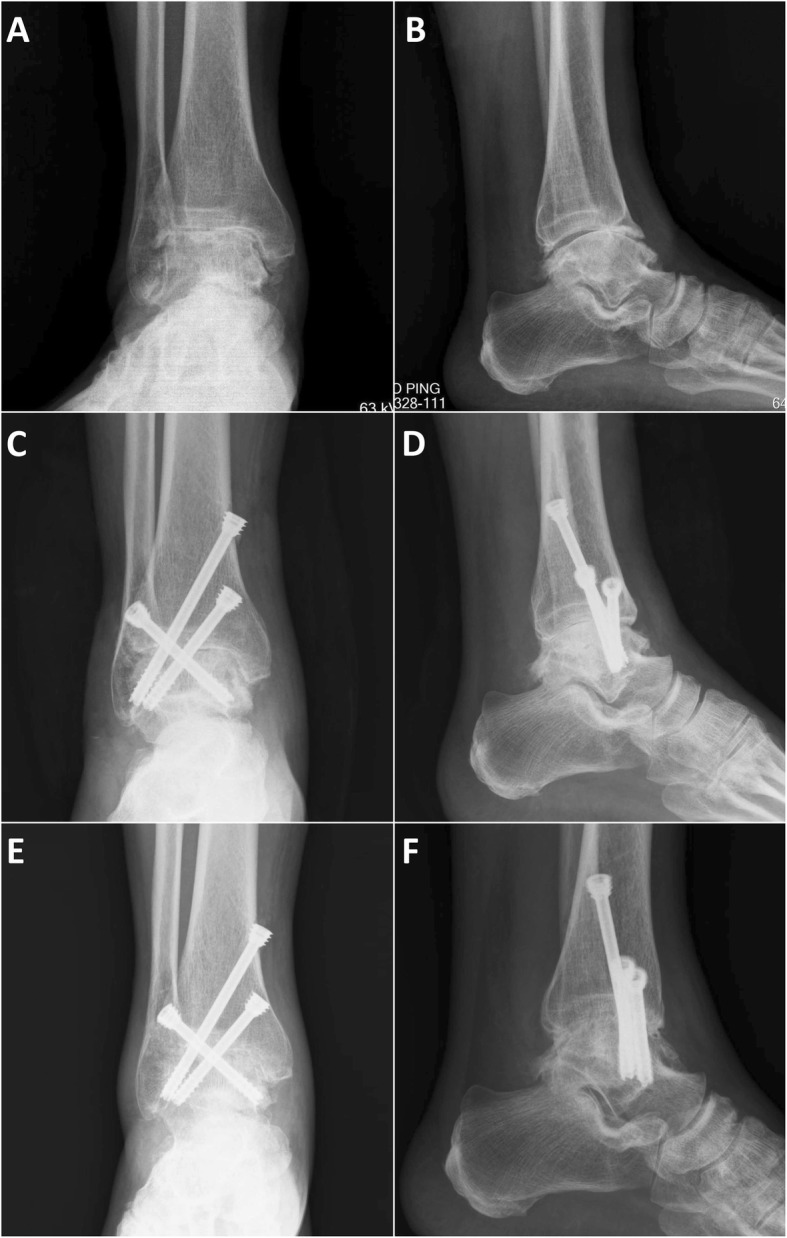
A typical case of AAF group, male, 63 years, the patient had a history of repeatedly sprained the right ankle for 8 years, and the ankle was found to be arthritis for 4 years. **a**, **b** Anteroposterior and lateral X-ray film 5 days before operation, we found that the orientation of ankle mortise can be acceptable. **c**, **d** Anteroposterior and lateral X-ray film 2 days after operation, we made an arthroscopic ankle fusion by using three large cannulated screws; the joint space was filled and pressurized. **e**, **f** Anteroposterior and lateral X-ray film 3 months after operation, no obvious internal fixation loosening was observed, and a lot of bone bridges were found in the joint space, the ankle fusion was ideal

**Fig. 2:**
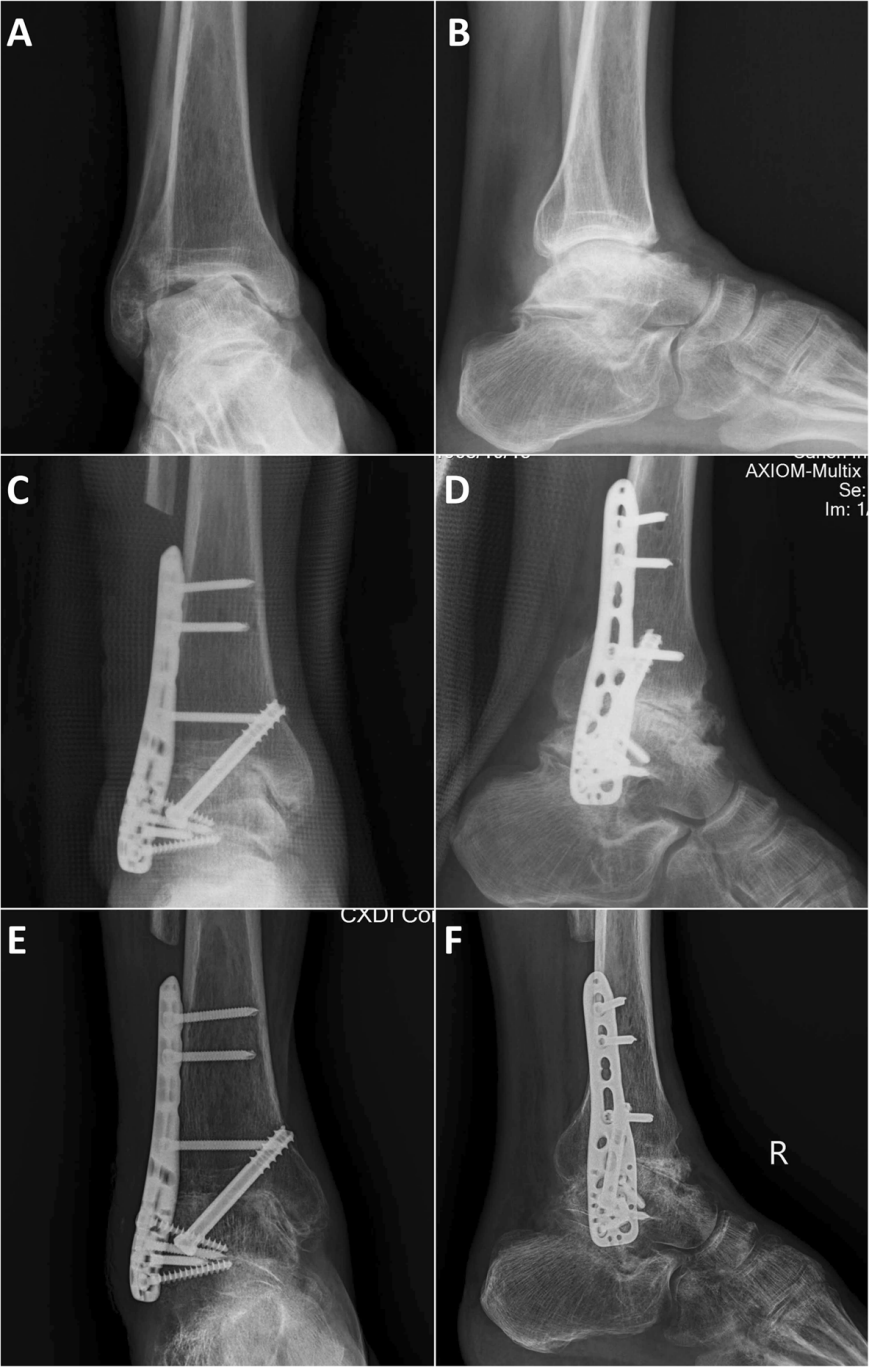
A typical case of COO subgroup, male, 49 years, the patient had a history of talus fractures 10 years ago, and followed by aseptic necrosis talus with ankle arthritis for 5 years. **a**, **b** Anteroposterior and lateral X-ray film 3 days before operation, we can found that the talus was severely varus and collapsed, and was hardened with a lot of sequestrum. **c**, **d** Anteroposterior and lateral X-ray film 3 days after operation; we made distal fibular osteotomy and large lesion cleaning, the distal fibula was separated and crushed, for impacted and structural bone grafting, the joint space was filled and pressurized. **e**, **f** Anteroposterior and lateral X-ray film 3 months after operation; no obvious internal fixation loosening was observed, and a small number of bone bridges were found in the joint space. However, the ankle fusion was not ideal at this point, and the patient had been found a good fusion 6 months after operation

**Fig. 3:**
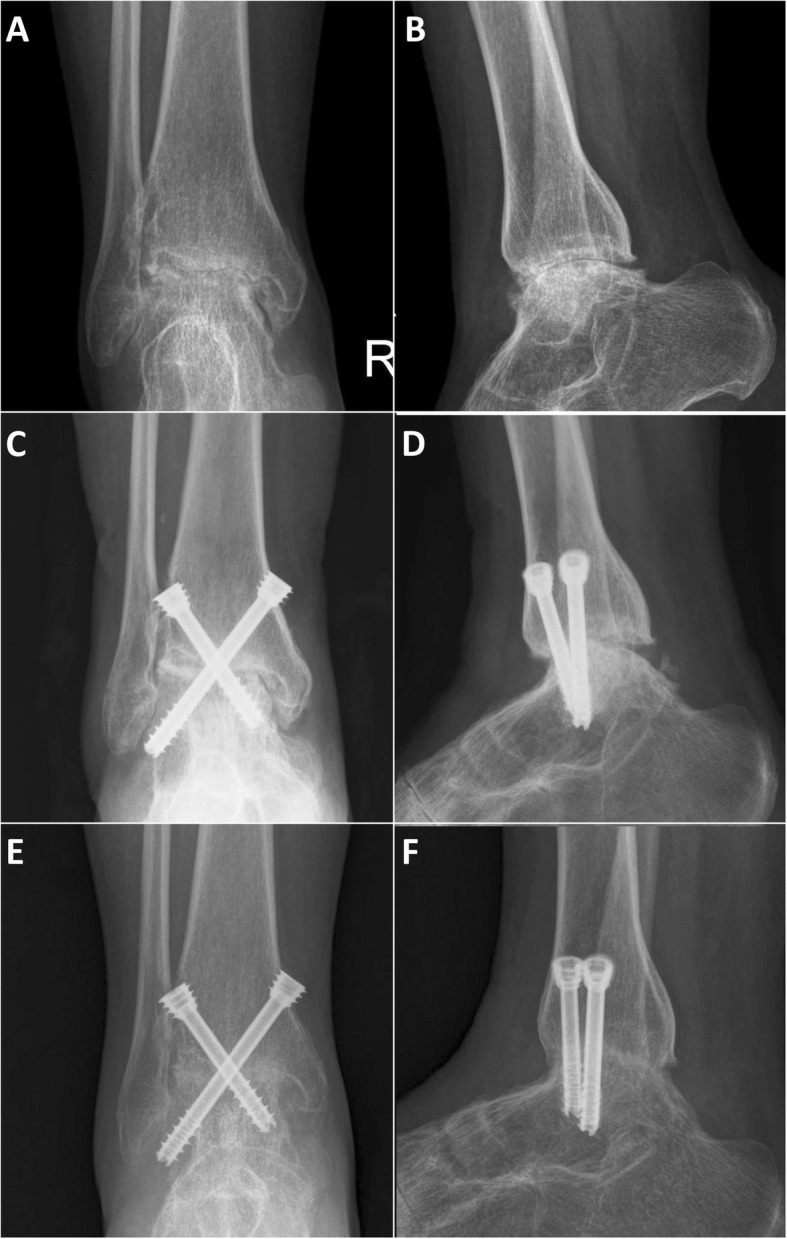
A typical case of SOO subgroup, female, 40 years, the ankle was found to be rheumatoid arthritis for 10 years. **a**, **b** Anteroposterior and lateral X-ray film 4 days before operation; we found that no obvious distortion of the ankle mortise orientation, but the joint space has disappeared and osteoporosis can be found. **c**, **d** Anteroposterior and lateral X-ray film 2 days after operation; we made an ankle fusion with an anterior approach by using two large cannulated screws; the joint space was pressurized. **e**, **f** Anteroposterior and lateral X-ray film 3 months after operation, no obvious internal fixation loosening was observed, and a lot of bone bridges were found in the joint space, the ankle fusion was ideal

By carefully searching the system with our study design, we divided the patients into two groups by surgical method: arthroscopic ankle fusion group (AAF group) (*n* = 17) (Fig. 1a, b); and open ankle fusion group (OAF group) (*n* = 26). The purpose of this study was to analyze the effect of surgical methods on ankle fusion only. In order to eliminate selection bias, we divided OAF group into two subgroups, which were used for more detailed comparison with AAF group. The OAF group was divided into complex osseous operation subgroup (COO subgroup) (*n* = 15) (Fig. 2a, b) and simple osseous operation subgroup (SOO subgroup) (*n* = 11) (Fig. 3a, b). The inclusion criteria of COO subgroup was the OAF cases whose osseous operation types which could not be available in AAF cases, such as distal fibula was separated or splitted, need more than 8 cm^3^ impacted bone graft and need structural bone graft. The inclusion criteria of SOO subgroup was the OAF cases whose osseous operation type was the same as the AAF group.

Basic information of the patients is presented in Table 1. Forty-three patients were enrolled in our study and received final follow-up; the demographics and preoperative situation of two groups have no significant difference. All patients were followed up 3 months, 6 months, and 1 year after surgery, and accepted at least one follow-up after postoperative 1 year again. Mean follow-up time: 33.7 months (range 14 months to 49 months)

**Table 1:** Patients demographics and preoperative conditions

	Between-group difference (all patients)			Difference detailed into subgroup (osseous operation type: not same)			Difference detailed into subgroup (osseous operation type: same)			Internal difference of OAF group (osseous operation type: not same)		
	AAF	OAF	P	AAF	COO	P	AAF	SOO	P	COO	SOO	*P*
Number of patients	17	26		17	15		17	11		15	11	
Mean of age (years) (95% CI)	54.76 ± 14.11	55.35 ± 12.52	0.891	54.76 ± 14.11	53.33 ± 14.82	0.782	54.76 ± 14.11	58.09 ± 8.35	0.441	53.33 ± 14.82	58.09 ± 8.35	0.310
Gender (Male/Female)	10/7	16/10	0.859	10/7	11/4	0.388	10/7	7/4	0.799	11/4	7/4	0.597
Smoker (No. and %)	3 (17.6%)	7 (26.9%)	0.481	3 (17.6%)	4 (26.7%)	0.538	3 (17.6%)	3 (27.3%)	0.544	4 (26.7%)	3 (27.3%)	0.973
Diabetes (No. and %)	4 (23.5%)	3 (11.5%)	0.298	4 (25.3%)	1 (6.7%)	0.190	4 (23.5%)	2 (18.2%)	0.736	1 (6.7%)	2 (18.2%)	0.364
Mean of BMI	26.55 ± 5.23	28.93 ± 5.95	0.188	26.55 ± 5.23	29.21 ± 5.22	0.162	26.55 ± 5.23	28.55 ± 7.09	0.400	29.21 ± 5.22	28.55 ± 7.09	0.786
Mean of AOFAS score (Preoperative)	36.2 ± 13.5	32.5 ± 11.8	0.347	36.2 ± 13.5	30.7 ± 12.8	0.253	36.2 ± 13.5	34.8 ± 10.5	0.780	30.7 ± 12.8	34.8 ± 10.5	0.395
Mean of follow-up time (months)	31.94 ± 11.07	34.81 ± 9.41	0.368	31.94 ± 11.07	36.53 ± 8.49	0.202	31.94 ± 11.07	32.45 ± 10.47	0.904	36.53 ± 8.49	32.45 ± 10.47	0.284

*AAF* arthroscopic ankle fusion group, *OAF* open ankle fusion group (demographic composition: OAF=COO+SOO), *COO* complex osseous operation; SOO: simple osseous operation, *BMI* body mass index, *AOFAS* score the American Orthopedic Foot and Ankle Society score. *P P* values: α = 0.05, (Age, BMI, AOFAS score and follow-up time: independent-samples t-test; gender, smoker and diabetes: Chi-squared test)

### Operative technique

#### Arthroscopic ankle fusion group

The operations were performed under sciatic and femoral nerve block anesthesia with additional general anesthesia. All patients were used two surgical entries located in front of the ankle space, medial to tibialis anterior tendon, and lateral to peroneus tertius tendon. A small number of patients have added posterior ankle entries on either side of the achilles tendon if necessary. We used arthroscopic burrs and soft-tissue shavers to remove synovium, cartilage, and osteonecrotic areas. After that, we made the subchondral bone to bleed heavily. The medial and lateral gutter joint surfaces were also denuded. We fused the ankle to a plantar flexion of 90°, ectropion of 5°, external of 5°, and make sure the ankle axis in an ideal position. We checked the position by anteroposterior and lateral intraoperative fluoroscopic images and direct vision until we are satisfied. According to the specific bone defect of joint space, we used pressure bone grafting by using excess osteophytes, proximal tibia bone graft (PTBG), or demineralized bone matrix (DBM). Fixation was achieved with two or three large cannulated screws (6.5 mm, 6.9 mm, and 7.2 mm) under fluoroscopic guidance (Fig. 1). The first screw entered from the lateral fibula or lateral tibial about 15–25 mm above the ankle line and penetrated the collum tali to the caput tali. The second screw entered from the medial tibia about 15–25 mm above the ankle line and penetrated to the area close to subtalar joint of the lateral talus. If we needed to install the third screw, its entry point was 5–10 mm higher than the second screw in general and penetrated to the area close to subtalar joint of the lateral talus too. We ensured the adequate screw fixation and compression across the fusion site under intraoperative fluoroscopic images and direct physical examination. We closed the wounds with simple interrupted nonabsorbable monofilament sutures.

#### Open ankle fusion group

In COO subgroup (*n* = 15), a transfibular approach was used in the majority of patients (13/15) (Fig. 2). In the transfibular approach, we made a curvilinear incision over the lateral ankle and removed the distal fibula. For majority of patients (*n* = 9), the distal fibula was separated or crushed, for impacted or structural bone grafting; and for other patients (*n* = 4), the distal fibula was split into two half portions longitudinally, the medial portion was used for bone grafting, and the lateral portion was retained for reconstruction by using screws. Then we exposed the tibiotalar joint and remove synovium, cartilage, and osteonecrotic areas. After that, we made the subchondral bone to bleed heavily with a 1.5–2.0 mm drill. The ankle was then provisionally pinned in position after the medial gutter joint surfaces were also denuded. We fused the ankle to a plantar flexion of 90°, ectropion of 5°, external of 5°, and make sure the ankle axis is in an ideal position. In most instances (*n* = 9), we placed one or two guidewires for cannulated screws (6.5 mm, 6.9 mm, and 7.2 mm). Then we installed the cannulated screws, the first screw entered from the area close to subtalar joint of the lateral talus and penetrated to the medial tibia about 15–25 mm above the ankle line, or the installation trajectory was just the opposite. If we needed to install the second screw, its entry point was located at the lateral tibia about 15–25 mm above the ankle line and penetrated the collum tali to the caput tali. We placed a locking plate laterally with three or four 3.5-mm screws placed into the talus and three or four screws placed proximally into the distal tibia. For other patients (*n* = 4), the procedures for joint cleaning and bone grafting were the same as above, but the tibial talus joint was fused by using two or three large cannulated screws (6.5 mm, 6.9 mm, and 7.2 mm) similar to AAF group; finally, the lateral portion of distal fibula mentioned above was secured to the lateral malleolus by two 3.5-mm screws. The remaining patients underwent either an anterior (*n* = 2) approach to use previous incisions. In this approach, we did not remove the distal fibula after the ankle articular cavity was exposed.

In SOO subgroup (*n* = 11), patients were treated with an anterior (*n* = 5), bilateral anterior-oblique (*n* = 4), anteromedial (*n* = 1), or anterolateral (*n* = 1) approach. In these approaches, we did not remove the distal fibula too. The joint was cleaned and fixed temporarily to the above position. The ankles were fixed by using two (*n* = 5) or three (*n* = 6) cannulated screws similar to the AAF group (Fig. 3)

### Postoperative management

After surgery, the patients of two groups were required to raise the limb, and the use of antibiotics was necessary. For OAF group, drainage was usually postoperatively. For both two groups, the patient wore a plaster slab until the stitch of wound was removed immediately after surgery, and then wore a below-knee protective plaster cast. Patients were encouraged to mobilize non-weight bearing for the first 4–6 weeks; after that, partially weight bearing for the next 4–8 weeks according to the doctor’s advice, if a partial union was seen at X-ray; afterwards, full weight bearing according to the doctor’s advice, if more than 30% of the cross section of firm bone bridge was seen at X-ray.

### Measurement and follow-up

We carefully searched and collected the following data through the medical records and follow-up system: surgical time, intra-op blood loss, reduction of albumin (ALB, the difference of albumin value between preoperative 2 days and postoperative 2 days), total hospital stays, union time, fusion situation, and complications; the American Orthopedic Foot and Ankle Society (AOFAS) ankle and hindfoot score [17, 21]; and the outcomes of follow-up questionnaire which referred to Musculoskeletal Outcomes Data Evaluation and Management Scale (MODEMS) questionnaire [19]. We got these data through outpatient or visiting services.

### Statistical analysis

After all the results of each time point of follow-up have been collected, we made statistical analyses of the data: independent Samples *t* test was used to assess the difference of the age, body mass index (BMI), follow-up time, AOFAS score, surgical time, intra-op blood loss, reduction of ALB, total hospital stays, union time, and questionnaire outcomes. Chi-squared test was used to analyze the difference of the gender, smoker, diabetes, and etiology. Statistical analyses were performed using SPSS 20.0 software (SPSS Inc., Chicago, IL, USA). *P* values less than 0.05 were considered statistically significant.

## Results

### Etiology and composition

By analyzing the etiological components of each group, we found that the etiological components of AAF group and SOO subgroup were similar, mainly including osteoarthritis, post-trauma arthritis, and infectious arthritis; SOO group was treated rheumatoid arthritis and urarthritis patients; the difference was that the etiology of COO group included talus necrosis, clubfoot, and charcot arthritis. The number and proportion of osteoarthritis (seven cases, 41.2%) (*P* = 0.008) and post-trauma arthritis cause by ligament lesions (five cases, 29.4%) (*P* = 0.018) of AAF group was significantly more than the OAF group (two cases, 7.7%; one cases, 3.8%); the number and proportion of talus necrosis of AAF group (0 case) was significantly less than the OAF group (four cases, 15.4%) (*P* = 0.038). Similarly, the number and proportion of osteoarthritis (seven cases, 41.2%) (*P* = 0.005) and post-trauma arthritis cause by ligament lesions (five cases, 29.4%) (*P* = 0.022) of AAF group was significantly more than the COO subgroup (0 case; 0 case); the number and proportion of talus necrosis of AAF group (0 case) was significantly less than the COO subgroup (four cases, 26.7%) (*P* = 0.023). The number and proportion of talus necrosis of COO subgroup (four cases, 26.7%) was significantly more than the SOO subgroup (0 case) (*P* = 0.026). (Table 2)

**Table 2:** Patients etiology conditions

	Between-group difference (all patients)			Difference detailed into subgroup (osseous operation type: not same)			Difference detailed into subgroup (osseous operation type: same)			Internal difference of OAF group (osseous operation type: not same)		
	AAF (*n* = 17)	OAF (*n* = 26)	*P*	AAF (*n* = 17)	COO (*n* = 15)	*P*	AAF (*n* = 17)	SOO (*n* = 11)		COO (*n* = 15)	SOO (*n* = 11)	*P*
Etiology (No. and %)												
Osteoarthritis	7 (41.2%)	2 (7.7%)	0.008	7 (41.2%)	0	0.005	7 (41.2%)	2 (18.2%)	0.203	0	2 (18.2%)	0.086
Post-trauma arthritis (fracture)	3 (17.6%)	11 (42.3%)	0.092	3 (17.6%)	7 (46.7%)	0.077	3 (17.6%)	4 (36.4%)	0.264	7 (46.7%)	4 (36.4%)	0.599
Posttrauma arthritis (ligament lesions)	5 (29.4%)	1 (3.8%)	0.018	5 (29.4%)	0	0.022	5 (29.4%)	1 (9.1%)	0.201	0	1 (9.1%)	0.234
Infectious arthritis	2 (11.8%)	2 (7.7%)	0.653	2 (11.8%)	1 (6.7%)	0.621	2 (11.8%)	1 (9.1%)	0.823	1 (6.7%)	1 (9.1%)	0.819
Rheumatoid arthritis	0	2 (7.7%)	0.242	0	0	NS	0	2 (18.2%)	0.068	0	2 (18.2%)	0.086
Urarthritis	0	1 (3.8%)	0.413	0	0	NS	0	1 (9.1%)	0.206	0	1 (9.1%)	0.234
Talus necrosis	0	4 (15.4%)	0.038	0	4 (26.7%)	0.023	0	0	NS	4 (26.7%)	0	0.026
Clubfoot	0	2 (7.7%)	0.242	0	2 (13.3%)	0.120	0	0	NS	2 (13.3%)	0	0.128
Charcot arthritis	0	1 (3.8%)	0.413	0	1 (6.7%)	0.279	0	0	NS	1 (6.7%)	0	0.288

*AAF* arthroscopic ankle fusion group, *OAF* open ankle fusion group (demographic composition: OAF = COO + SOO), *COO* complex osseous operation, *SOO* simple osseous operation. *P P* values: α = 0.05 (Chi-squared test)

### Operation outcomes

**Fig. 4:**
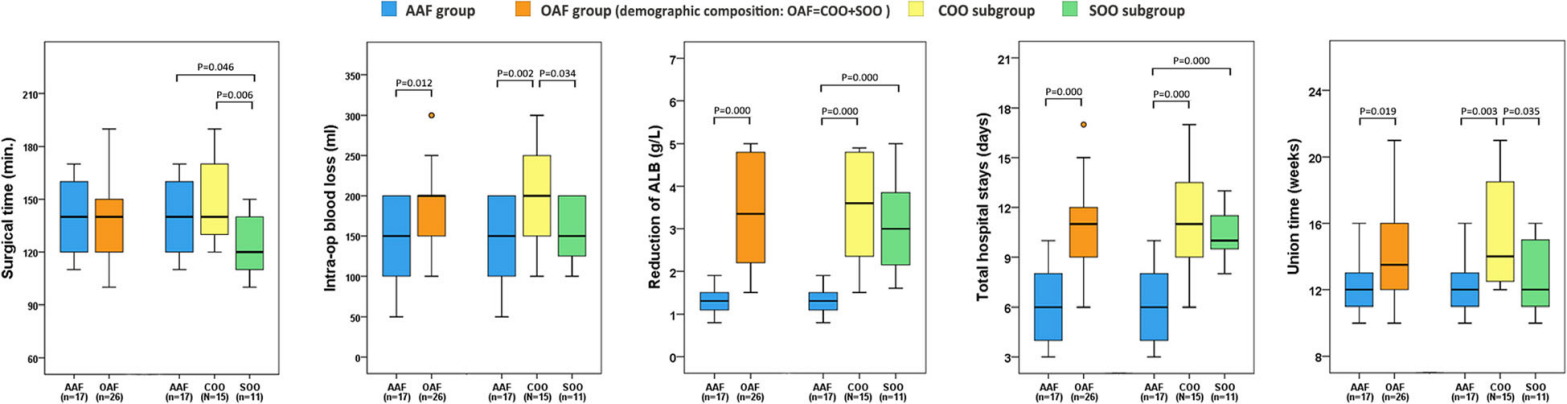
Box plot of surgical time, intra-op blood loss, reduction of ALB, total hospital stays, and union time: We can found that the surgical time of SOO subgroup was significantly less than the AAF group and COO subgroup. The intra-op blood loss and the union time of AAF group were significantly less than the OAF group and the COO subgroup, and the SOO subgroup were significantly less than the COO subgroup too. The reduction of ALB and the total hospital stays of AAF group were significantly less than the OAF group, the COO subgroup and the SOO subgroup

Through postoperative imaging examination, all groups can be found the joint space were filled and pressurized (Figs. 1, 2, and 3) The surgical time of SOO subgroup (123.6 ± 18.6 min) was significantly less than the AAF group (140.5 ± 22.2 min) (*P* = 0.046) and COO subgroup (149.3 ± 23.1 min) (*P* = 0.006). The intra-op blood loss of AAF group (137.1 ± 49.7 ml) was significantly less than the OAF group (184.6 ± 62.9 ml) (*P* = 0.012) and the COO subgroup (206.7 ± 67.8 ml) (*P* = 0.002), and the intra-op blood loss of SOO subgroup (154.5 ± 41.6 ml) was significantly less than the COO subgroup (206.7 ± 67.8 ml) (*P* = 0.034). The reduction of ALB (the difference of albumin value between preoperative 2 days and postoperative 2 days) (1.28 ±0.28 g/L) of AAF group was significantly less than the OAF group (3.35 ± 1.19 g/L) (*P* = 0.000), the COO subgroup (3.49 ± 1.22 g/L) (*P* = 0.000), and the SOO subgroup (3.15 ± 1.19 g/L) (*P* = 0.000). Similarly, the total hospital stays of AAF group (6.3 ± 2.5 days) was significantly less than the OAF group (11.0 ± 2.7 days) (*P* = 0.000), the COO subgroup (11.4 ± 3.4 days) (*P* = 0.000), and the SOO subgroup (10.5 ±1.5 days) (*P* = 0.000). One patient of AAF group, two patients of COO subgroup, and one patient of SOO subgroup had transient paralysis of nerve after operation; three patients of COO subgroup and one patient of SOO subgroup had delayed wound healing after operation; and one patient of COO subgroup had soft tissue infection after operation (Table 3, Fig. 4).

**Table 3:** Outcomes of perioperative, fusion, and complications situation.

	Between-group difference (all patients)			Difference detailed into subgroup (osseous operation type: not same)			Difference detailed into subgroup (osseous operation type: same)			Internal difference of OAF group (osseous operation type: not same)		
	AAF (*n* = 17)	OAF (*n* = 26)	*P*	AAF (*n* = 17)	COO (*n* = 15)	*P*	AAF (*n* = 17)	SOO (*n* = 11)	*P*	COO (*n* = 15)	SOO (*n* = 11)	*P*
Mean of surgical time (min.)	140.5 ± 22.2	138.5 ± 24.6	0.775	140.5 ± 22.2	149.3 ± 23.1	0.284	140.5 ± 22.2	123.6 ± 18.6	0.046	149.3 ± 23.1	123.6 ± 18.6	0.006
Mean of intra-op blood loss (ml)	137.1 ± 49.7	184.6 ± 62.9	0.012	137.1 ± 49.7	206.7 ± 67.8	0.002	137.1 ± 49.7	154.5 ± 41.6	0.343	206.7 ± 67.8	154.5 ± 41.6	0.034
Mean of reduction of ALB * (g/L)	1.28 ± 0.28	3.35 ± 1.19	0.000	1.28 ± 0.28	3.49 ± 1.22	0.000	1.28 ± 0.28	3.15 ± 1.19	0.000	3.49 ± 1.22	3.15 ± 1.19	0.474
Mean of total hospital stays (days)	6.3 ± 2.5	11.0 ± 2.7	0.000	6.3 ± 2.5	11.4 ± 3.4	0.000	6.3 ± 2.5	10.5 ± 1.5	0.000	11.4 ± 3.4	10.5 ± 1.5	0.396
Mean of union time (weeks)	12.4 ± 1.9	14.6 ± 3.4	0.019	12.4 ± 1.9	15.5 ± 3.5	0.003	12.4 ± 1.9	12.8 ± 2.3	0.563	15.5 ± 3.5	12.8 ± 2.3	0.035
No fusion (No. and %)	0	2 (7.7%)		0	2 (13.3%)		0	0		2 (13.3%)	0	
Complications situation	AAFgroup: transient paralysis of nerve (one case) SCsubgroup: delayed wound healing (three cases); transient paralysis of nerve (two cases); infection (one case) NSCsubgroup: delayed wound healing (one case); transient paralysis of nerve (one case)											

*AAF* arthroscopic ankle fusion group, *OAF* open ankle fusion group (demographic composition: OAF = COO + SOO), *COO* complex osseous operation, *SOO* simple osseous operation; reduction of ALB: The difference of albumin value between preoperative 2 days and postoperative 2 days. *P P* values: α = 0.05, (Independent-samples *t* test)

### Follow-up outcomes

At 3 months after the operation, all patients were followed up in the outpatient and completed the AOFAS score. At 6 months and 1 year after the operation, there were respectively three and four patients lost to follow-up, but the proportion in each group was less than 15%. At the final follow-up, all patients were followed up and completed the AOFAS score and questionnaire survey (Table 4)

**Table 4:** Outcomes of AOFAS score

	Between-group difference (all patients)			Difference detailed into subgroup (osseous operation type: not same)			Difference detailed into subgroup (osseous operation type: same)			Internal difference of OAF group (osseous operation type: not same)		
	AAF	OAF	P	AAF	COO	P	AAF	SOO	P	COO	SOO	P
Mean of AOFAS score												
Within 2 weeks before surgery	36.2 ± 13.5 (*n* = 17)	32.5 ± 11.8 (*n* = 26)	0.347	36.2 ± 13.5 (*n* = 17)	30.7 ± 12.8 (*n* = 15)	0.253	36.2 ± 13.5 (*n* = 17)	34.8 ± 10.5 (*n* = 11)	0.780	30.7 ± 12.8 (*n* = 15)	34.8 ± 10.5 (*n* = 11)	0.395
Post-op 3 months	63.1 ± 3.3 (*n* = 17)	59.6 ± 4.7 (*n* = 26)	0.013	63.1 ± 3.3 (*n* = 17)	57.9 ± 4.7 (*n* = 15)	0.001	63.1 ± 3.3 (*n* = 17)	61.9 ± 3.9 (*n* = 11)	0.412	57.9 ± 4.7 (*n* = 15)	61.9 ± 3.9 (*n* = 11)	0.031
Post-op 6 months	70.5 ± 5.7 (*n* = 15)	66.7 ± 5.1 (*n* = 25)	0.034	70.5 ± 5.7 (*n* = 15)	64.9 ± 3.8 (*n* = 15)	0.004	70.5 ± 5.7 (*n* = 15)	69.4 ± 5.7 (*n* = 10)	0.633	64.9 ± 3.8 (*n* = 15)	69.4 ± 5.7 (*n* = 10)	0.027
Post-op 1 year	76.2 ± 3.9 (*n* = 16)	73.0 ± 7.1 (*n* = 23)	0.160	76.2 ± 3.9 (*n* = 16)	72.1 ± 6.2 (*n* = 14)	0.037	76.2 ± 3.9 (*n* = 16)	74.3 ± 8.1 (*n* = 9)	0.439	72.1 ± 6.2 (*n* = 14)	74.3 ± 8.1 (*n* = 9)	0.467
Final (at least 1 year post-op)	77.7 ± 3.8 (*n* = 17)	75.8 ± 4.5 (*n* = 26)	0.148	77.7 ± 3.8 (*n* = 17)	75.4 ± 3.7 (*n* = 15)	0.090	77.7 ± 3.8 (*n* = 17)	76.3 ± 5.6 (*n* = 11)	0.423	75.4 ± 3.7 (*n* = 15)	76.3 ± 5.6 (*n* = 11)	0.632

*AAF* arthroscopic ankle fusion group, *OAF* open ankle fusion group (demographic composition: OAF = COO + SOO), *COO* complex osseous operation, *SOO* simple osseous operation, *Post*-*op* postoperative, *AOFAS* score the American Orthopedic Foot and Ankle Society score. *P P* values: α = 0.05 (Independent-samples *t* test)

#### Radiological and fusion examination

At 3 months after operation, no obvious internal fixation loosening were observed in all groups; for most of the AAF group and SOO subgroup patients, we can find a lot of bone bridges in the joint space, and the ankle fusion were ideal; but there were only a part of patients in COO subgroup can be found ideal ankle fusion (Fig. 1, 2, and 3) The union time of AAF group (12.4 ± 1.9 weeks) was significantly less than the OAF group (14.6 ± 3.4 weeks) (*P* = 0.019) and the COO subgroup (15.5 ± 3.5 weeks) (*P* = 0.003), and the union time of SOO subgroup (12.8 ± 2.3 weeks) was significantly less than the COO subgroup (15.5 ± 3.5 weeks) (*P* = 0.035). In COO subgroup, there were two cases that had non-fusion after primary ankle fusion surgical; both of them underwent refusion revision surgery (Table 3, Fig. 4).

#### Functional score

**Fig. 5:**
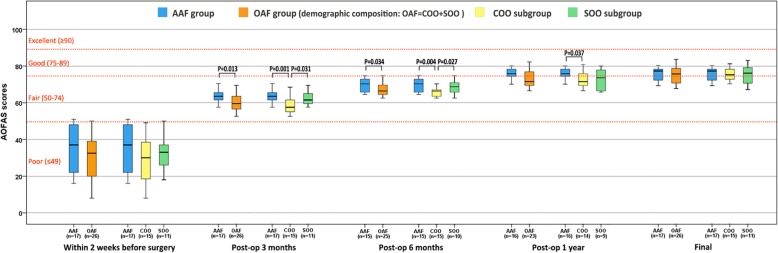
Box plot of AOFAS scores at every time points: The curative effect and recovery rate of AAF group and SOO subgroup were both better than the COO subgroup after operation until one year after operation; there was no significant difference between these three groups from one year after operation, and the AOFAS score mean of each group was greater than 75 points at that time point

By analyzing the results of AOFAS scores, the curative effect and recovery rate of AAF group and SOO subgroup were both better than the COO subgroup after operation until 1 year after operation: The postoperative 3-month AOFAS score of AAF group (63.1 ± 3.3) was significantly better than the OAF group (59.6 ± 4.7) (*P* = 0.013) and the COO subgroup (57.9 ±4.7) (*P* = 0.001), and the postoperative 3-month AOFAS score of SOO subgroup (61.9 ± 3.9) was significantly better than the COO subgroup (57.9 ± 4.7) (*P* = 0.031). The postoperative 6-month AOFAS score of AAF group (70.5 ± 5.7) was significantly better than the OAF group (66.7 ± 5.1) (*P* = 0.034) and the COO subgroup (64.9 ± 3.8) (*P* = 0.004), and the postoperative 6-month AOFAS score of SOO subgroup (69.4 ± 5.7) was significantly better than the COO subgroup (64.9 ± 3.8) (*P* = 0.027). The postoperative 1-year AOFAS score of AAF group (76.2 ± 3.9) was significantly better than the COO subgroup (72.1 ± 6.2) (*P* = 0.037). But there was no significant difference between these three groups from one year after operation, and the AOFAS score mean of each group was greater than 75 points (Table 4, Fig. 5).

#### Questionnaire survey

**Fig. 6:**
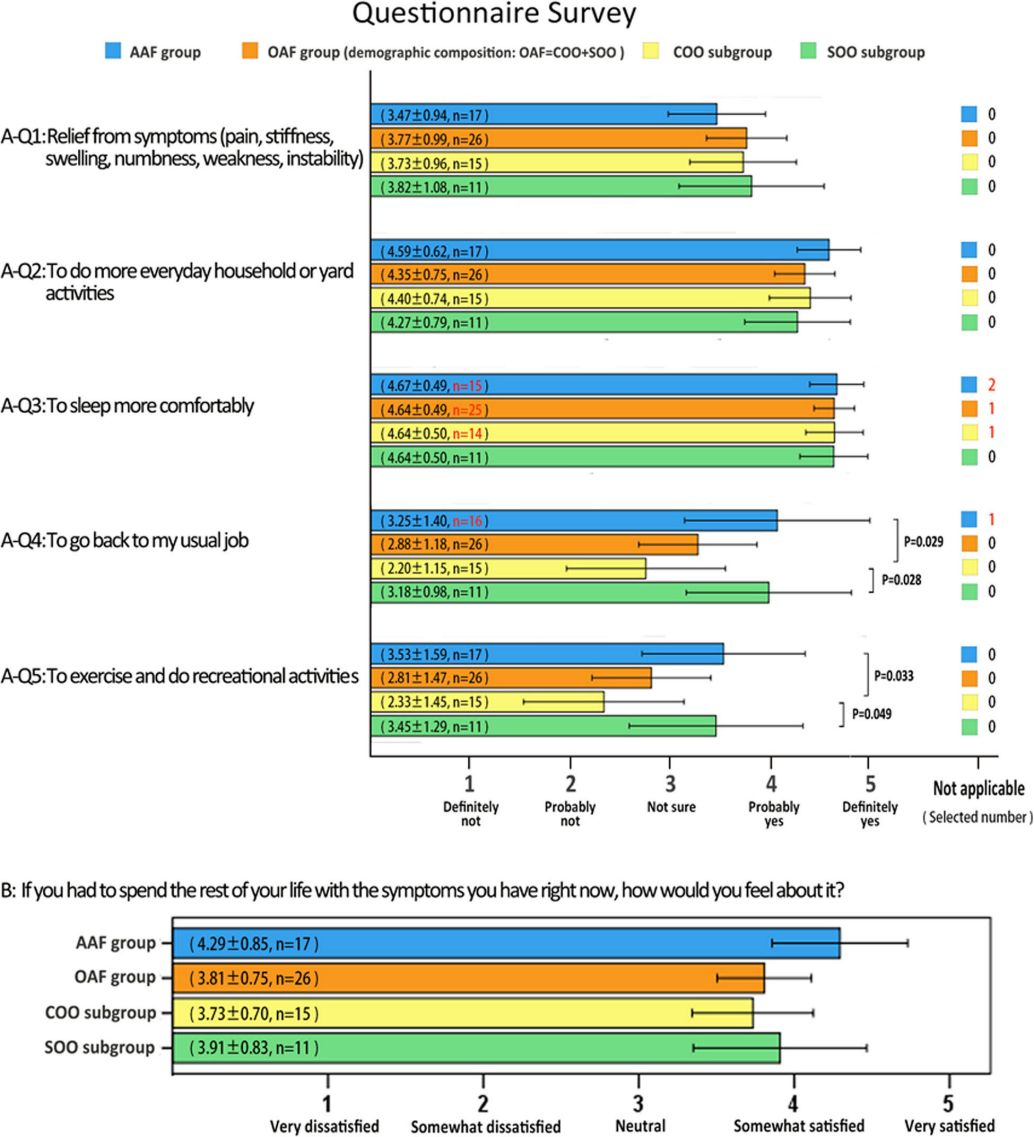
Outcomes of questionnaire survey: The survey was conducted during the final follow-up of the study. The questionnaire referred to Musculoskeletal Outcomes Data Evaluation and Management Scale (MODEMS) questionnaire, and each question was single choice. The case label of bar chart uses a 95% confidence interval

The results of questionnaire survey (Fig. 6) shows: first, no matter which groups were not satisfied with the improvement of the pain and other symptoms through question A-1. Second, each patient gave a high score to question A-2 and A-3, which suggested that almost all patients can do some mellow work without walking long-distance, and can sleep well. Third, when asked about participating in usual job and recreational activities, almost all the patients of AAF group and SOO subgroup gave significantly better answers than the COO subgroup. Four, overall, the patients felt somewhat satisfied if they had to spend the rest of their life with the symptoms they have at the final follow-up of the study.

## Discussion

Although there were many studies considered, the arthroscopic ankle fusion achieve better rates of fusion and better follow-up outcomes than open fusion. However, these studies usually ignored the influence of two aspects on the results. On the one hand, some patients need complex osseous operation with poor curative effect could only choose open surgery and the comparison results are also likely to favor arthroscopic ankle fusion method, thus selection bias happened; on the other hand, the judgment of the efficacy of ankle fusion should be combined with subjective evaluation, which was also lacking in relevant studies. So we conducted a retrospective comparative study: first, the OAF group, which was regrouped according to the osseous operation type; second, we used MODEMS questionnaire during follow-up. It is worth noting that some different results seemed to be found, and we discuss them as follows.

### About etiology

By statistical analysis, we found that the etiological components of AAF group and OAF group were different, and the etiological components of AAF group and SOO subgroup were similar, mainly including the cases not needing osteotomy, orthopedics, large lesion cleaning, or large bone graft reconstruction, such as osteoarthritis, post-trauma arthritis caused by ligament lesions, and rheumatoid arthritis [22, 23]. However, the etiological components of AAF group and COO subgroup were significantly different, such as advanced talus necrosis, severe clubfoot, and charcot arthritis, because the osseous operation types of COO subgroup were different from AAF group and SOO subgroup; and the surgical effect of COO subgroup was poor [24–26]. This also supports the correctness of the grouping of subgroup for OAF group to some extent. We believed that such experimental design of further grouping can improve the credibility. After all, roughly dividing patients into AAF group and OAF group according to surgical approach would lead to selection bias, especially for retrospective studies [27, 28]. Therefore, this study compared the AAF group with the COO subgroup and the SOO subgroup respectively; it would be easier to exclude the selection bias caused by the severity of the disease, so as to objectively analyze the differences caused by surgical methods.

### About operation

Through postoperative imaging examination, all groups can be found the joint space were filled and pressurized; it means that the arthroscopic ankle fusion technology can deal with the joint surface well when the osseous operation type is the same as SOO subgroup. The surgical time of SOO subgroup was the fastest compared with other groups; it means that AAF group has no advantage in surgical time compared with OAF group, because the clearance of joint surface is very complicated and slow by arthroscopic technology. AAF group and SOO subgroup have obvious advantages in intra-op blood loss compared with COO subgroup; the possible reason is that the increase in surgical time cancels out the advantage of fewer traumas compared with SOO subgroup. However, it is worth noting that AAF group has obvious advantages in avoiding ALB reduction, total hospital stays, and avoiding complications compared with other groups; the reason can be explained by the small trauma of AAF group. Some studies considered that arthroscopic ankle fusion can result in lower complication rate and shorter hospital stay as compared to open-ankle fusions [29, 30].

### About radiological and fusion

At 3 months after operation, for most of the AAF group and SOO subgroup patients, we can find a lot of bone bridges in the joint space, and the ankle fusion were ideal; but there were only a part of patients in COO subgroup where ideal ankle fusion can be found. The union time of AAF group and SOO subgroup were significantly less than the OAF group and the COO subgroup. In COO subgroup, there were two cases occurred nonfusion after primary ankle fusion surgical; both of them underwent refusion revision surgery. This shows that the fusion time of AAF group which similar to that of SOO subgroup is faster than COO subgroup until 1 year after the surgery, but has no advantage in long-term follow-up. The possible reason is the surgical trauma and illness condition of COO subgroup were more severe, and the osseous operation type was different. There are many similar studies; it is reported that the overall non-union rates after anterior arthroscopic assisted ankle fusions are 8.6% [31]; and some researches shows that the minimally invasive operation would have good fusion under the premise the cannulated screws are installed well [32, 33]. However, none of these studies took into account the selection bias of grouping caused by the difference of the osseous operation type, so it cannot be generally considered that the fusion condition of arthroscopic technology has an advantage over open technology.

### About functional score

By analyzing the results of AOFAS scores, the curative effect and recovery rate of AAF group and SOO subgroup were both better than the COO subgroup after operation until 1 year after operation, but there was no significant difference between these three groups from 1 year after operation, and the AOFAS score mean of each group was greater than 75 points, which can be considered that the function is good according to the current mainstream view. This shows that the scores of all groups failed to achieve excellent; the reason is the nature of ankle surgery itself. The gait of patients had still showed slight limp after ankle fusion, and the patients would appear in pain and swelling when they walk for a long time, which has been widely reported [18, 34–36]. Ebalard et al. reported that 84% of patients complained of pain after a minimum follow-up of 10 years [37]. In other studies, the prevalence of osteoarthrosis ranged from 24 to 100% in the subtalar joint and from 18 to 77% in the Chopart joints [38]. What is more notable is that the recovery condition of AAF group which is similar to that of SOO subgroup is faster than COO subgroup until 1 year after the surgery, but has no advantage in long-term follow-up. The possible reason is the osseous operation types of COO subgroup are more complex, so it cannot be generally considered that the function score of arthroscopic technology is better than open technology.

### *About questionnaire survey*

Patient expectation and satisfaction may be valuable measures for defining the success of an operative intervention [39, 40], so we designed a part of questionnaire survey in our study Additional file 1. The expectation scores used in the present study were referred to the MODEMS scores, which are currently the only scores that are applicable to ankle outcomes [19]. In the part of questionnaire survey, through question A-1, we can see no matter which groups were not satisfied with the improvement of the pain and other symptoms; the possible reason was the gait of patients had still been showed with slight limp after ankle fusion, and the patients would appear in pain and swelling when they walk for a long time, which lead to a bad subjective feeling of the patients. Notably, dissatisfaction with symptoms appeared to be higher in the AAF group, possibly because these patients had a higher expectation because they thought the minimally invasive surgery would help them get a faster recovery. Each patient gave a high score to question A-2 and A-3, which suggested that almost all patients can do some mellow work without walking long-distance, and can sleep well; but it is worth noting that there are three individuals who chose N/A in question A-3, which reflected that the diseases that need ankle fusion have little effect on sleep even before operation. When asked about participating in usual job and recreational activities, almost all the patients of AAF group and SOO subgroup gave significantly better answers than the COO subgroup; the possible reason should be that the patients in the COO subgroup thought it was difficult to return to usual job and recreational activities due to the severe disease, poor surgical effect, and fair subjective effect. Finally, almost all the patients felt somewhat satisfied if they had to spend the rest of their life with the symptoms they have at the final follow-up of the study; this result shows that although ankle fusion cannot make the patients obtain excellent functions, it can still help the patients in all groups get recovery to some extent and make them satisfied. Even more remarkable, question A-2 and A-3 revealed some differences that were not demonstrated by AOFAS score; the possible reason is the patient’s expectations may therefore be quite different from the actual result of function in many case; it is also worth noting that this discrepancy is a potential source of patient dissatisfaction [41, 42].

## Limitation

Firstly, the follow-up time of the study is still not long enough to observe the complications such as degeneration of the surrounding joint. Secondly, it is necessary to research the survival rate of ankle joint in each group after surgery on the basis of long-term observation in the future. Thirdly, this study is only a retrospective study. A prospective study which excludes confounding factors by good experimental design should be conducted, even randomized controlled trial.

## Conclusions

In summary, we found that AAF group has advantages over OAF group in surgical procedure, fusion condition, and postoperative recovery in general. However, after the selection bias was minimized from the grouping of OAF group again, which was divided into two subgroups according to the osseous operation type, we found that AAF group only has advantages over COO subgroup. It is worth noting that except in terms of surgical trauma, hospital stays and short-term complications occurred, the AAF group has no obvious advantages over SOO subgroup, which disease condition and operation method are similar to AAF group, including intra-op blood loss, fusion condition, postoperative function score, and postoperative patient satisfaction; and AAF group need more surgical time than the SOO subgroup. Therefore, we believe that the arthroscopic ankle fusion can bring a good curative effect; however, if the osseous operation type is the same, the arthroscopic ankle fusion only has a limited advantage over the traditional open operation in perioperative soft tissue protection and enhanced recovery after surgery.

## Supplementary information

**Additional file 1.** Presentation of the questionnaire contents.

## Data Availability

The datasets used and/or analyzed during the study are available from the corresponding author upon reasonable request.

## Abbreviations

AAF: Arthroscopic ankle fusion
ALB: Albumin
AOFAS: American Orthopedic Foot and Ankle Society
BMI: Body mass index
COO: Complex osseous operation
DBM: Demineralized bone matrix
MODEMS: Musculoskeletal Outcomes Data Evaluation and Management Scale
OAF: Open ankle fusion
PTBG: Proximal tibia bone graft
SOO: Simple osseous operation
