# Supplementary figures and images for: Arthroscopic ankle fusion only has a limited advantage over the open operation if osseous operation type is the same: a retrospective comparative study
Source: J Orthop Surg Res. 2020 Feb 26;15:80. doi: 10.1186/s13018-020-01599-5 (PMC7045598; doi:10.1186/s13018-020-01599-5)

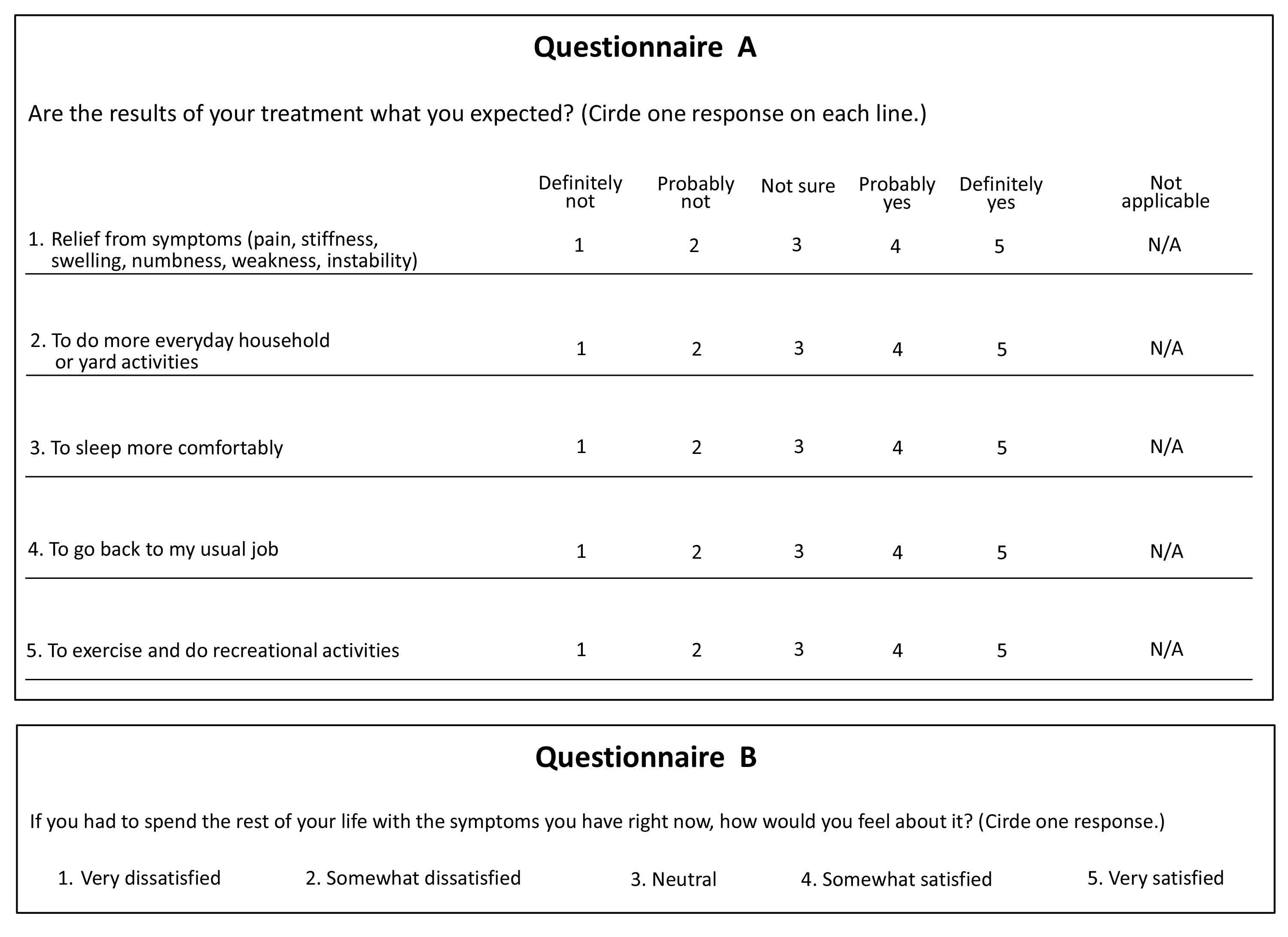

Supplement: Supplementary file 1 — Additional file 1. Presentation of the questionnaire contents. [file 13018_2020_1599_MOESM1_ESM.jpg]
